# Both 3,3′,5-triiodothyronine and 3,5-diodo-L-thyronine Are Able to Repair Mitochondrial DNA Damage but by Different Mechanisms

**DOI:** 10.3389/fendo.2019.00216

**Published:** 2019-04-09

**Authors:** Federica Cioffi, Rosalba Senese, Giuseppe Petito, Pasquale Lasala, Pieter de Lange, Elena Silvestri, Assunta Lombardi, Maria Moreno, Fernando Goglia, Antonia Lanni

**Affiliations:** ^1^Dipartimento di Scienze e Tecnologie, Università degli Studi del Sannio, Benevento, Italy; ^2^Dipartimento di Scienze e Tecnologie Ambientali, Biologiche e Farmaceutiche, Università degli Studi della Campania Luigi Vanvitelli, Caserta, Italy; ^3^Dipartimento di Biologia, Università degli Studi di Napoli Federico II, Naples, Italy

**Keywords:** mitochondria, liver, mtDNA, oxidative damage, iodothyronines

## Abstract

This study evaluated the effect of 3,5-diiodo-L-thyronine (T2) and 3,5,3′-triiodo-L-thyronine (T3) on rat liver mitochondrial DNA (mtDNA) oxidative damage and repair and to investigate their ability to induce protective effects against oxidative stress. Control rats, rats receiving a daily injection of T2 (N+T2) for 1 week and rats receiving a daily injection of T3 (N+T3) for 1 week, were used throughout the study. In the liver, mtDNA oxidative damage [by measuring mtDNA lesion frequency and expression of DNA polymerase γ (POLG)], mtDNA copy number, mitochondrial biogenesis [by measuring amplification of mtDNA/nDNA and expression of peroxisome proliferator-activated receptor gamma co-activator 1-alpha (PGC-1α)], and oxidative stress [by measuring serum levels of 8-hydroxy-2′-deoxyguanosine (8-OHdG)] were detected. T2 reduces mtDNA lesion frequency and increases the expression of POLG, and it does not change the mtDNA copy number, the expression of PGC-1α, or the serum levels of 8-OHdG. Therefore, T2, by stimulating the major mtDNA repair enzyme, maintains genomic integrity. Similar to T2, T3 decreases mtDNA lesion frequency but increases the serum levels of 8-OHdG, and it decreases the expression of POLG. Moreover, as expected, T3 increases the mtDNA copy number and the expression of PGC-1α. Thus, in T3-treated rats, the increase of 8-OHdG and the decrease of POLG indicate that there is increased oxidative damage and that the decreased mtDNA lesion frequency might be a consequence of increased mitochondrial biogenesis. These data demonstrate that both T2 and T3 are able to decrease in the liver mtDNA oxidative damage, but they act via different mechanisms.

## Introduction

Currently, scientists are paying increasing attention to mitochondria. Indeed, these organelles hold a key position in several crucial cellular processes, such as cell respiration, lipid metabolism, apoptosis, and ATP-synthesis. In view of these roles, scientists are increasingly paying attention to the integrity of these organelles and to the causes that could damage them, such as reactive oxygen species (ROS). In fact, mitochondria are considered the major source of endogenous reactive oxygen species, involving some components of the respiratory chain and redox enzymes (1). Moreover, these organelles contain components that make up an effective system of protection against oxidizing molecules (2, 3), making mitochondria important players in cellular redox homeostasis.

Increases in mitochondrial ROS production are associated with some pathological conditions (4) such as Alzheimer's and Parkinson's diseases (5), cancer (6), and ischaemia–reperfusion injury (7). Several altered physiological conditions may affect mitochondrial cellular redox homeostasis, and, among others, thyroid hormones (THs) seem to have a profound influence. Indeed, it is a widely accepted concept that thyroid hormones regulate metabolism and markedly affect mitochondrial respiration. Alteration of the thyroid state (hypothyroid or hyperthyroid state) results in an alteration of mitochondrial respiration, being decreased in hypothyroidism and increased in hyperthyroidism (8).

However, increased mitochondrial oxygen consumption corresponds to an increased production of mitochondrial oxygen radicals that damage mitochondria. These radicals can damage macromolecules in mitochondria, including proteins, lipids, RNA, and DNA (9). Information about the control of tissue oxidative stress by THs are scarce. It has been shown that variations in H_2_O_2_ flux of mitochondrial origin can induce oxidative nuclear DNA damage and may constitute an important part of endogenous damage to DNA (10). However, early studies by Richter et al. suggested that mitochondrial DNA (mtDNA) might be more prone to oxidative damage than nuclear DNA (nDNA) (11).

In addition, studies of the effects of THs often reveal conflicting data, and these inconsistencies can be attributed to the hormonal treatment employed (dosage, route of administration, duration, and strategy for inhibition of the hormones/thyroid gland), the animal species studied and the tissue assayed (8).

T3 is the active form of THs, but in recent years, among TH derivatives, T2 has been shown to exert marked effects on energy metabolism by acting mainly at the mitochondrial level. In liver, T2 administration prevented high fat diet (HFD)-induced lipid peroxidation and increased mitochondrial H_2_O_2_ metabolism, in addition, it reduced the upregulation of both PPAR-alpha and Mitofusin 2 (MT-2). These data demonstrate that the administration of T2 in HFD rats prevents both lipid accumulation in the liver and oxidative stress associated with increased fat metabolism (12, 13).

From previous studies, we believe it is interesting to investigate the effects that both T3 and T2 exert on oxidative damage of mtDNA and their repair mechanisms. To maintain genomic integrity, different DNA repair pathways have evolved, and the pathways employed depend, in part, upon the type of DNA damage that is being repaired. In particular, simpler lesions such as alkylation or oxidation products caused by ROS are repaired by the base excision repair (BER) pathway (11). Several studies indicate that one of the best studied and major repair mechanisms in the mitochondria is that of BER. Oxidative DNA damage is largely repaired by the BER pathway, so it is fitting that this pathway pre-dominates in an organelle thought to have evolved to protect against oxygen toxicity (14–17). Consequently, to have further indications about the state of oxidative stress, we also measured the serum levels of 8-OHdG. 8-OHdG is by far the most sensitive biomarker for determination of oxidative DNA damage and has gained much attention because of its mutagenic potential (18). The cellular defense system against 8-OHdG mutagenesis involves BER, nucleotide excision repair, mismatch repair and prevention of incorporation (19). Because of this, in this study, we evaluate the effect of T3 and T2 on mtDNA oxidative damage and repair by the BER pathway in the liver of euthyroid rats.

## Materials and Methods

### Animals and Treatments

Male Wistar rats (250–300 g; Charles River, Lecco, Italy) were kept one per cage in a temperature-controlled room at 28°C (thermoneutral temperature for rat), with a 12-h light-dark cycle, with free access to food (commercial mash) and water. Three groups were used, with each group including four animals: (1) N, euthyroid control rats sham injected; (2) N+T2, euthyroid rats receiving a daily i.p administration of T2 (25 μg/100 g b.w.) for 1 week; (3) N+T3, euthyroid rats receiving a daily i.p administration of T3 (15 μg/100 g b.w. for 1 week) for 1 week. The dose of T3 was chosen to induce a slight hyperthyroidism in order to promote the production of free radicals. In fact, the serum levels of T3 were: TT3 (nmol/l) 1.01 ± 0.005; 0.99 ± 0.006 and 2.03 ± 0.050 for N, N+T2, and N+T3, respectively. Instead, the dose of 25 μg of T2 was chosen as it was the minimum dose capable of inducing metabolic effects without causing deleterious side effects (20–22). It was not possible to evaluate its serum levels because there are currently no commercially available kits.

All animal protocols were approved by the Committee on the Ethics of Animal Experiments of the University of Campania “Luigi Vanvitelli,” (Italy) and the Italian Minister of Health. Every effort was made to minimize animal pain and suffering. At the end of the treatment, rats were anesthetized by an i.p. injection of chloral hydrate (40 mg/100 g bodyweight) and killed by decapitation. The livers were immediately dissected, weighted, after which they were either processed for the isolation of mitochondria or immediately frozen in liquid and nitrogen and later stored at −80°C until processed.

### Genomic DNA Isolation

The total liver DNA was extracted using the Genomic-tip 20/G kit (Qiagen, Valencia, CA, USA) according to the manufacturer's protocol. The quantification of the purified genomic DNA and PCR products was performed fluorometrically using the PicoGreen dsDNA reagent (Invitrogen, Milan, Italy).

### Quantitative PCR (QPCR)

QPCR was performed as previously described (9). Tissues were ground in liquid nitrogen, and total DNA was extracted using the Genomic-tip 20/G kit (Qiagen) according to the manufacturer's protocol. Two pairs of PCR primers were employed:

mtDNA long fragment (13.4 kb): 5′-AAAATCCCCGCAAACAATGACCACCC-3′ (sense) and 5′-GGCAATTAAGAGTGGGATGGAGCCAA-3′ (anti-sense);

mtDNA short fragment (235 bp): 5′-CCTCCCATTCATTATCGCCGCCCTGC-3′ (sense) and 5′-GTCTGGGTCTCCTAGTAGGTCTGGGAA-3′ (anti-sense).

For amplification of the mtDNA long fragment, the standard thermocycler programme included initial denaturation at 94°C for 1 min, 18 cycles of 94°C for 15 s, 65°C for 12 min, and final extension at 72°C for 10 min. To amplify the short mtDNA fragment (235 bp), the same programme was used except the extension temperature was changed to 60°C. DNA damage was quantified by comparing the relative efficiency of amplification of the long mtDNA fragment normalized to the amplification of the small mtDNA fragment. QPCR products were quantified using PicoGreen dye and a fluorescence plate reader in the same manner as the template DNA. The resulting values were converted to relative lesion frequencies per 10 kb DNA by applying the Poisson distribution:

(1) $$\frac{lesions}{amplicon}=-ln\left(\frac{A_{d}}{A_{o}}\right)$$

where A_d_ represents amplification of treated samples, and A_o_ represents amplification of untreated controls.

### Quantification of mtDNA Copy Number by Real Time PCR

Relative mtDNA copy numbers were measured by real-time PCR and corrected by simultaneous measurement of nuclear DNA. We examined the amplification of mitochondrial cytochrome c oxidase subunit II (COII, mitochondrial-encoded gene) and β-actin (nuclear-encoded gene). The primer sequences used were as follows:

COII: 5′-TGAGCCATCCCTTCACTAGG-3′ (sense) and 5′-TGAGCCGCAAATTTCAGAG-3′ (anti-sense);

β-actin: 5′-CTGCTCTTTCCCAGATGAGG-3′ (sense) and 5′-CCACAGCACTGTAGGGGTTT-3′ (anti-sense).

The threshold cycle (Ct) reflects the cycle number at which a fluorescence signal within a reaction crosses a threshold. In our study, the average Ct values of nDNA and mtDNA were obtained for each case. mtDNA content was calculated using ΔCt = average Ct_nuclearDNA_-average Ct_mtDNA_ and was then obtained using the formula mtDNA content = 2(2ΔCt).

### RNA Isolation and Quantitative RT-PCR

The total liver RNA was isolated using TRIzol® reagent (Invitrogen) according to the manufacturer's protocol. Tissue/TRIzol® mixtures were homogenized using an Ultra-Turrax homogenizer, while keeping the viscosity of the solution to a minimum to ensure effective inactivation of endogenous RNase activity. The RNA samples were subjected to DNase treatment to remove genomic DNA contamination. A total of 1 μg of total RNA was used to generate cDNA in a 20 μL reaction volume using Superscript II Reverse Transcriptase (HT Biotechnology, Cambridge, UK). PCR primers were designed using Primer Express version 2.0 (Invitrogen). β-Actin mRNA expression was used for normalization. Primers used were as follows:

β-actin: 5′-CTGCTCTTTCCCAGATGAGG-3′ (sense) and 5′-CCACAGCACTGTAGGGGTTT-3′ (anti-sense);

POLG: 5′-GAAGAGCGTTACTCTTGGACCAG-3′ (sense) and 5′-AACATTGTGCCCCACCACTAAC-3′ (anti-sense);

PGC-1α: 5′-GTCAACAGCAAAAGCCACAA-3′ (sense) and 5′-GTGTGAGGAGGGTCATCGTT-3′ (anti-sense);

MnSOD: 5′-CCAAAGGAGAGTTGCTGGAG-3′(sense) and 5′-GAACCTTGGACTCCCACAGA-3′ (anti-sense).

An equivalent of 25 ng of total RNA was subsequently used in the amplification with 50 nM of gene-specific primers and 4 mL of iTaq Universal SYBR Green mix (Bio-Rad Laboratories, Hercules, CA, USA) in a total volume of 8 μL using standard cycle parameters on a Bio-Rad iQ5.

### Immunoassay for 8-OHdG

A competitive ELISA of 8-OHdG was performed using a DNA/RNA Oxidative Damage ELISA kit (Cayman Chemical Company, Ann Arbor, Michigan, USA) according to the manufacturer's protocol. Serum samples were analyzed in duplicate. Standard 8-OHdG was assayed over a concentration range of 10.3–3,000 pg/mL in duplicate for each experiment.

### Preparation of Total Protein Lysates

The liver tissue was homogenized in lysis buffer containing 20 mM Tris-HCl (pH 7.5), 150 mM NaCl, 1 mM EDTA, 1 mM EGTA, 2.5 mM Na_2_H_2_P_2_O_7_, 1 mM b-CH_3_H_7_O_6_PNa_2_, 1 mM Na_3_VO_4_, 1 mM PMSF, 1 mg/mL leupeptin, and 1% Triton X-100 (Sigma-Aldrich, St. Louis, MO, USA) using an Ultra-Turrax homogenizer, and then centrifuged at 15,000 × g in a Beckman Optima TLX Ultracentrifuge (Beckman Coulter S.p.A., Milan, Italy) for 15 min at 4°C. The supernatants were then ultracentrifuged at 40,000 × g in a Beckman Optima TLX ultracentrifuge for 15 min at 4°C. For determination of liver content, the supernatants were used without further processing. The protein concentration in supernatants and cleared lysates was determined using the Bio-Rad DC method.

### Preparation of Hepatic Homogenate and Isolated Mitochondria

Rat liver was gently homogenized in 10 volumes of isolation medium consisting of 220 mmol mannitol, 70 mmol sucrose, 20 mmol Tris-HCl, and 1 mmol EDTA at pH 7.4 (Sigma-Aldrich, St. Louis, MO, USA). The mitochondrial pellet was then washed twice and solubilized in a minimal volume of RIPA buffer [50 mmol Tris-HCl (pH = 7.4), 150 mmol NaCl, 1% NP-40, 0.1% SDS, 2 mmol EDTA, 0.5% sodium deoxycholate] until addition of protease/phosphatase inhibitors and kept on ice. The mitochondrial protein concentration was determined using the Bio-Rad DC method and the mitochondrial samples were then used for Western blot analysis of MnSOD content.

### Western BLOTTING

Western blot analysis was performed as described previously (23). The following antibodies were used: POLG (Novus Biologicals) and PGC-1α (Millipore). A β-actin antibody was purchased from Sigma-Aldrich. Manganese superoxide dismutase (MnSOD) protein levels were measured on protein extracts from isolated liver mitochondria using a polyclonal antibody (Santa Cruz Biotechnology) and a Ponceau S (Sigma-Aldrich), staining was used as mitochondrial loading control.

### Statistical Analysis

The results are expressed as the means ± SEM. The data were analyzed using one-way analysis of variance, *post-hoc* test: “Newman-Keuls Multiple Comparison Test.” Differences were considered statistically significant at *p* < 0.05.

## Results

### Effects of T2 and T3 on mtDNA Damage and Copy Number

**Figure 1:**
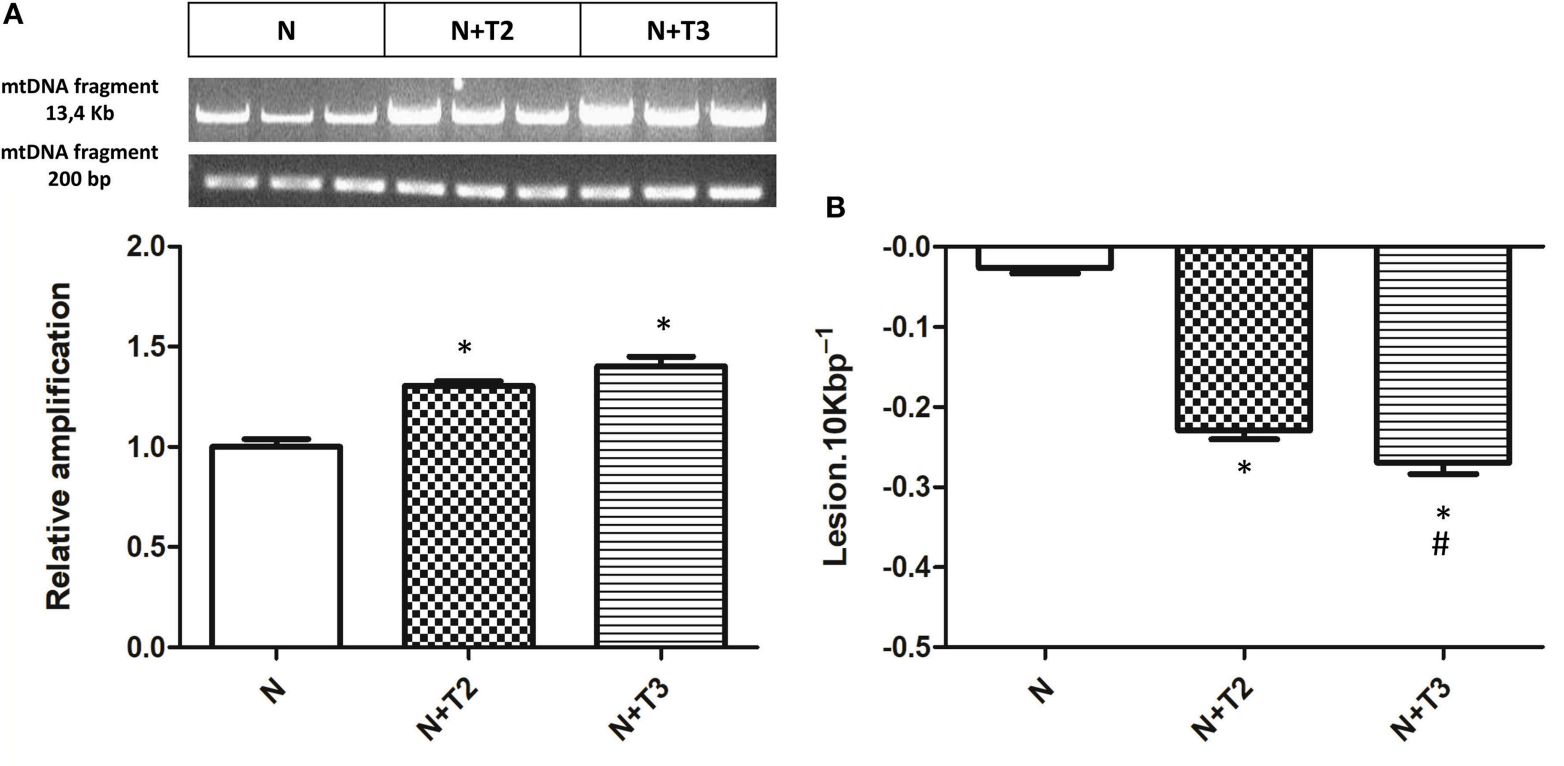
Effects of T2 and T3 on mtDNA damage and lesion frequency. **(A)** mtDNA damage was evaluated in the liver by amplifying long (13.4 kb) and short (235 bp) mtDNA fragments by QPCR; **(B)** Frequency of mtDNA lesions per 10 kb per strand. Values are presented as the means ± SEM from four rats in each group. ^*^p < 0.05 vs. N rats and #*p* < 0.05 vs. N+T2 rats. N: control rats; N+T2: rats receiving T2; N+T3: rats receiving T3.

**Figure 2:**
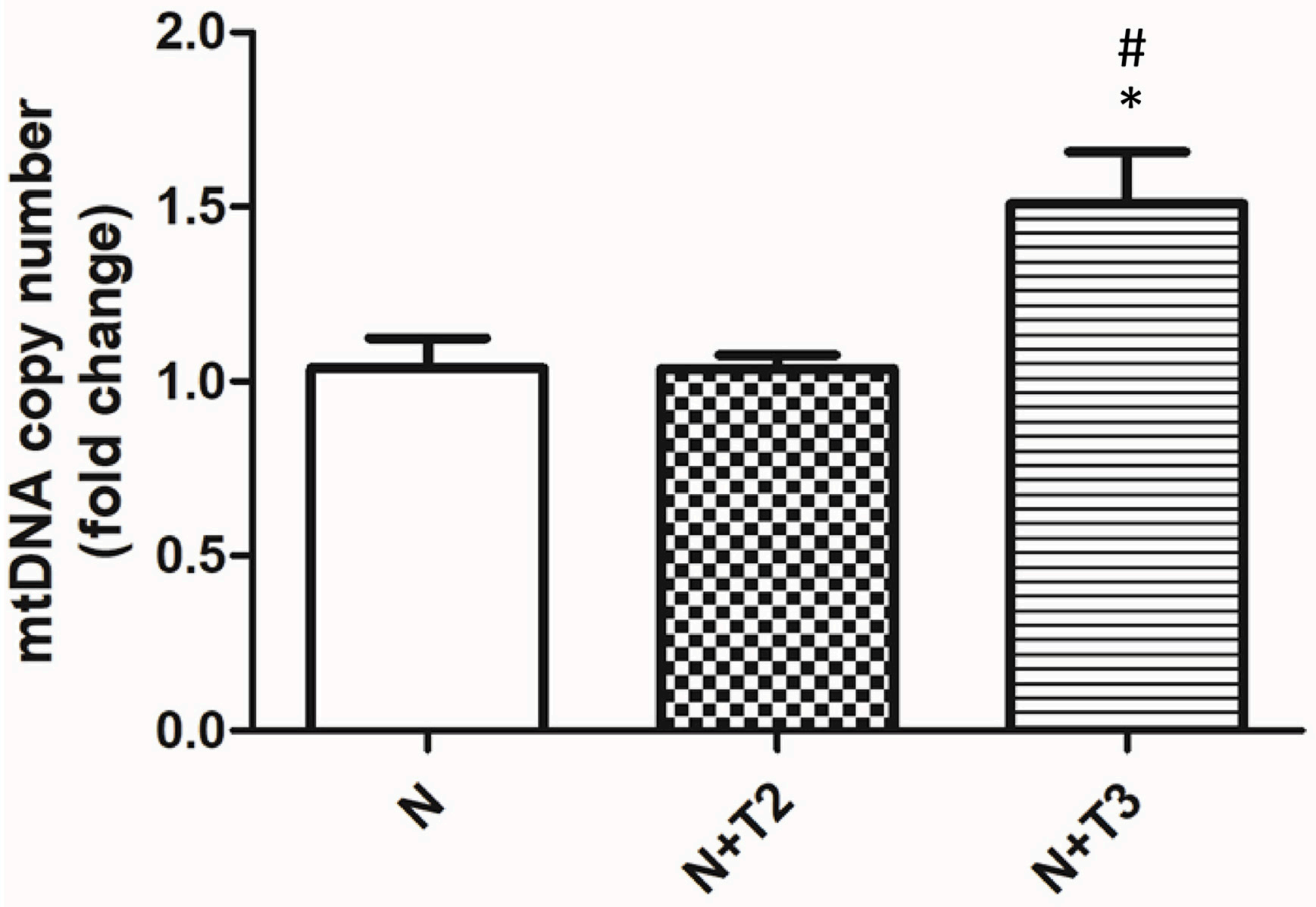
Effects of T2 and T3 on mtDNA copy number. mtDNA copy number was assessed by Real Time PCR in 10 ng of genomic liver DNA using primers for mtCOII. Expression was normalized using nuclear β-actin as an internal control. Values are presented as the means ± SEM from four rats in each group. ^*^p < 0.05 vs. N and #*p* < 0.05 vs. N+T2 rats. N: control rats; N+T2: rats receiving T2; N+T3: rats receiving T3.

Mitochondria are prominent target of oxidative damage and mtDNA is subject to severe oxidative damage, much more so than nDNA. QPCR was used to measure the levels of mtDNA oxidative damage. In N+T2 and N+T3 rats, the relative amplification of long (13.4 kb) and short (235 bp) mtDNA fragments was significantly increased by 30 and 40%, respectively, compared to control (N) rats (Figure 1A). Liver from N+T2 and N+T3 rats contained lower levels of mtDNA lesions compared to N rats (−0.23 and −0.27 lesions/10 kb/strand vs. −0.026 lesions/10 kb/strand, respectively; Figure 1B). These results demonstrate that N+T2 and N+T3 rats had significant decreases in liver mtDNA damage with T2 and T3 treatment. Moreover, the mtDNA copy number was significantly increased with T3 treatment but was not altered with T2 treatment, compared to N rats (Figure 2).

### Effects of T2 and T3 on 8-OHdG Levels in Serum

**Figure 3:**
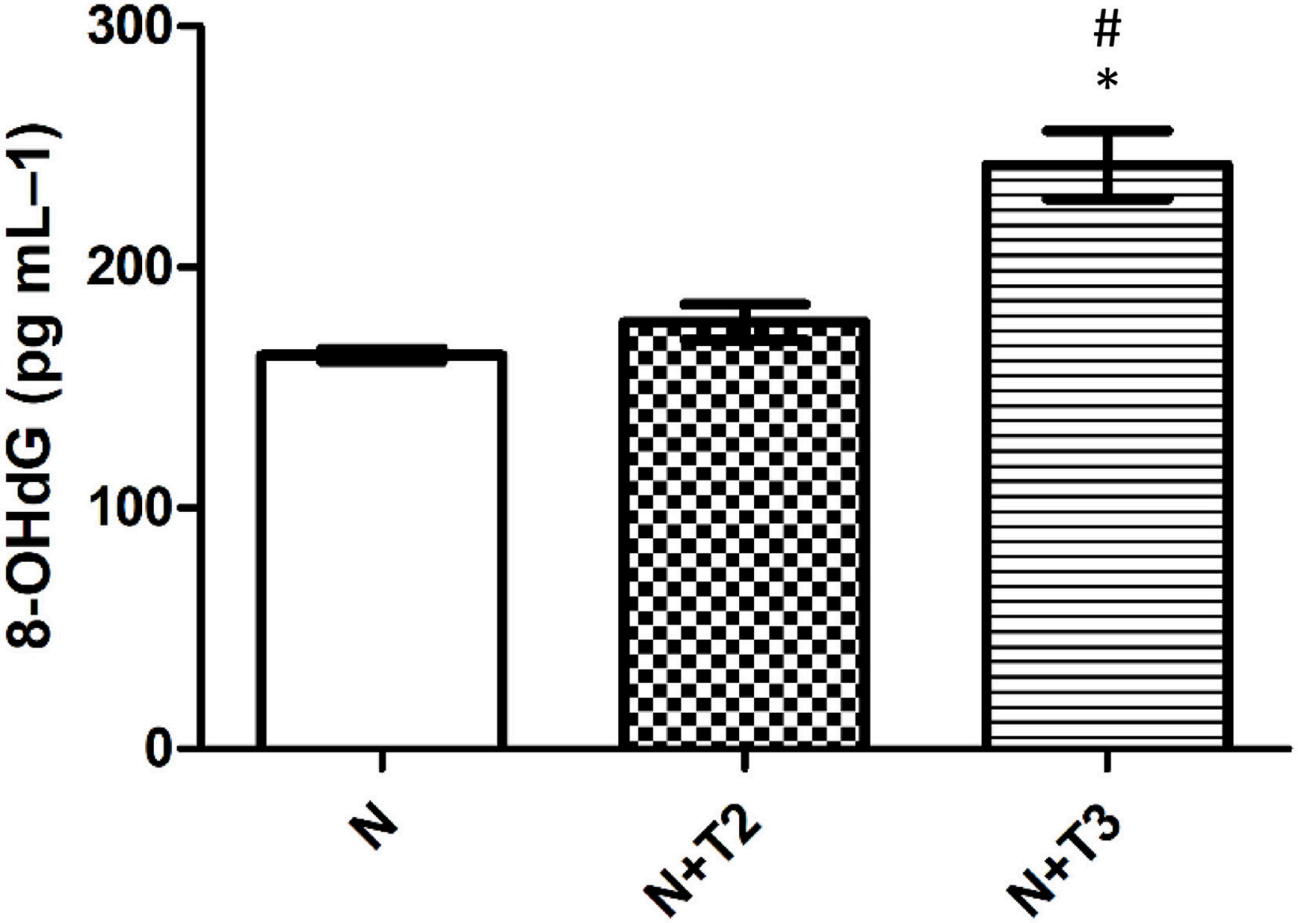
Effects of T2 and T3 on plasma 8-OHdG levels. Values represent means ± SEM from four rats in each group. ^*^p < 0.05 vs. N and #*p* < 0.05 vs. N+T2 rats. N: control rats; N+T2: rats receiving T2; N+T3: rats receiving T3.

Guanine is the base that is most prone to oxidation, and 8-OHdG from DNA is the form of oxidized guanine that is most commonly studied and is the main product of the oxidation to DNA. For this reason, 8-OHdG is considered a marker of oxidative stress. As shown in Figure 3, serum levels of 8-OHdG were significantly increased in the N+T3 rats compared to N, while there were no variations in N+T2 rats.

### Effect of T2 and T3 on MnSOD Expression

**Figure 4:**
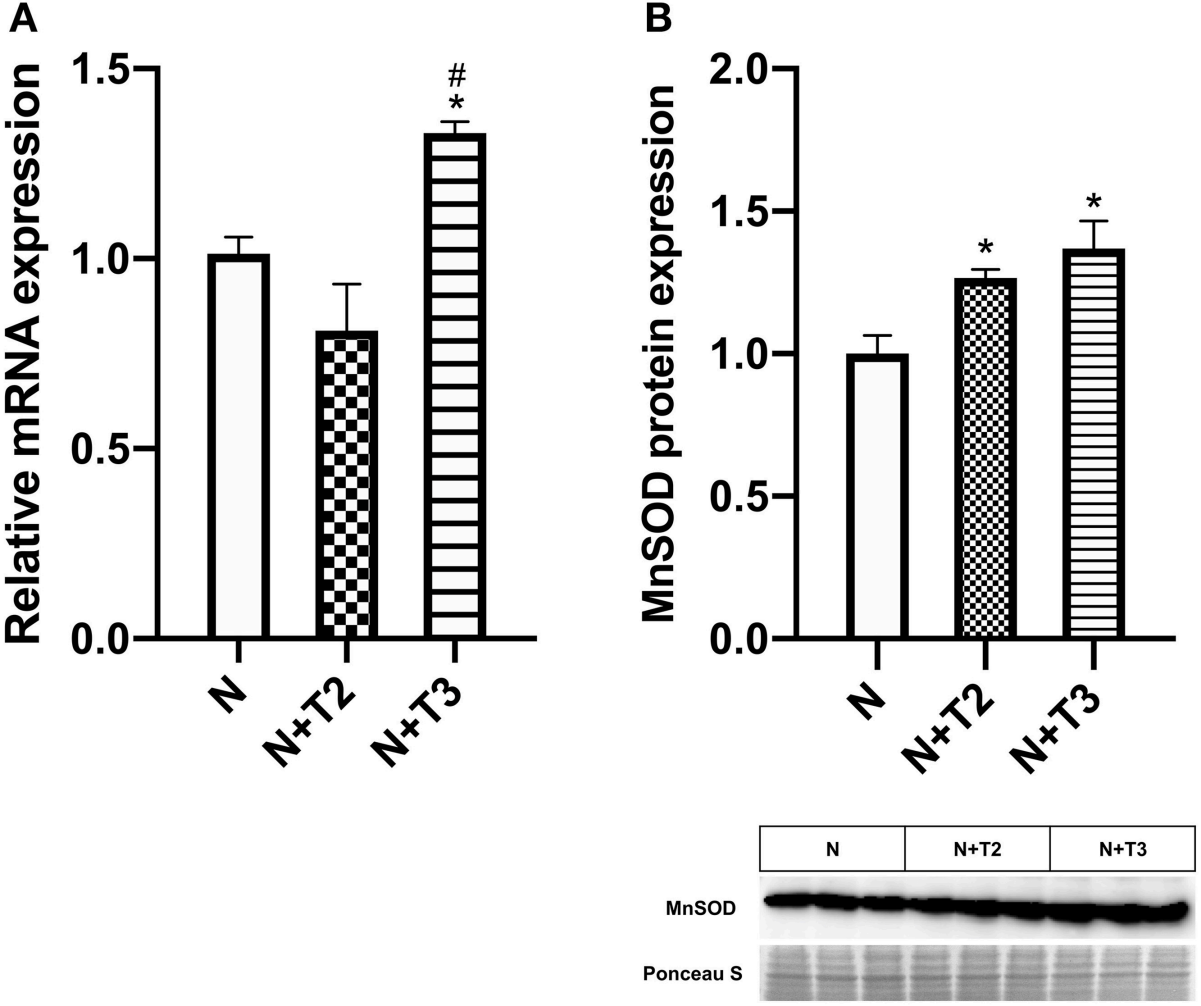
Effects of T2 and T3 on MnSOD expression. **(A)** MnSOD mRNA expression in liver tissues from different groups; **(B)** MnSOD mitochondrial protein expression in liver tissues from different groups and a representative result is shown. Values are presented as the means ± SEM from four rats in each group. ^*^p < 0.05 vs. N and #*p* < 0.05 vs. N+T2 rats. N: control rats; N+T2: rats receiving T2; N+T3: rats receiving T3.

MnSOD constitutes a major protective mechanism against ROS by removing them from mitochondria (24). Since it has been demonstrated that T_3_-induced pro-oxidant activity stimulated the expression of MnSOD, we indirectly confirm the oxidative stress induced by the iodothyronines by measuring the expression of MnSOD, qRT-PCR, and western blot analysis were performed. In N+T2 rats, MnSOD mitochondrial protein levels (Figure 4B) were found to be significantly increased by 27% compared to control rats, while no variation was detected in mRNA expression. In N+T3 rats, mRNA expression (Figure 4A) and mitochondrial protein levels of MnSOD (Figure 4B) were found to be significantly increased by 33 and 35%, respectively, compared to N rats.

### Effects of T2 and T3 on Mitochondrial Biogenesis

**Figure 5:**
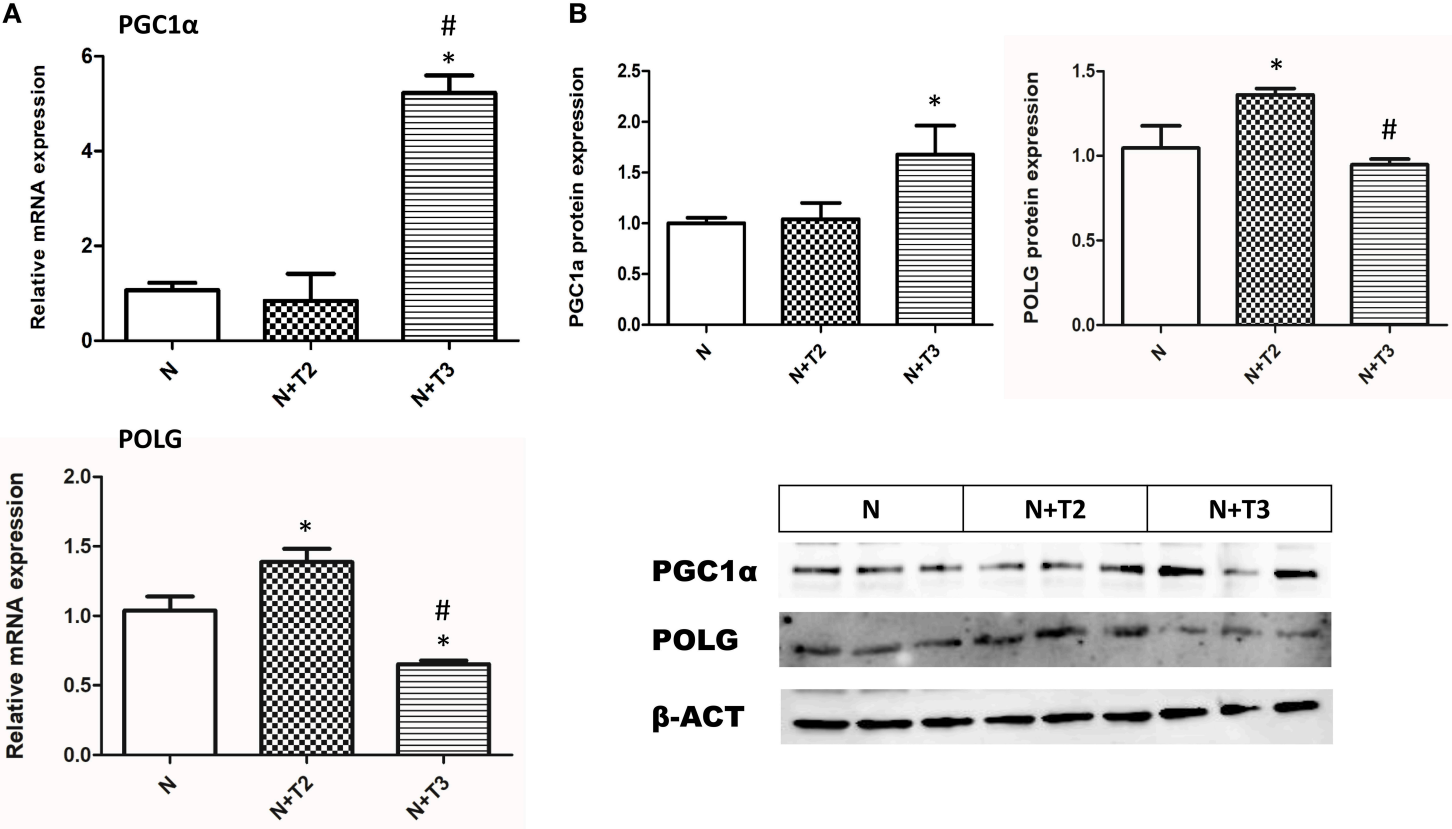
Effects of T2 and T3 on PGC-1α and POLG expression. **(A)** PGC-1α and POLG mRNA expression in liver tissues from different groups; **(B)** PGC-1α and POLG protein expression in liver tissues from different groups and a representative result is shown. Values are presented as the means ± SEM from four rats in each group. ^*^p < 0.05 vs. N and #*p* < 0.05 vs. N+T2 rats. N: control rats; N+T2: rats receiving T2; N+T3: rats receiving T3.

PGC1α is a central regulator of mitochondrial gene expression and is an essential component of mitochondrial biogenesis. We examined expression levels of PGC-1α by RT-PCR and western blot. As shown in Figures 5A,B, only T3 treatment increased mRNA and protein levels of PGC-1α compared to N and N+T2 rats.

### Effects of T2 and T3 on Mitochondrial POLG

POLG is an important enzyme involved in mtDNA repair and replication. To investigate whether T2 and T3 caused changes in POLG expression, qRT-PCR and western blot analysis were performed. In N+T2 rats, POLG mRNA expression (Figure 5A) and protein levels (Figure 5B) were found to be significantly increased by 44 and 27%, respectively, compared to control rats. While in N+T3 rats, POLG mRNA expression (Figure 5A) and protein levels (Figure 5B) were found to be significantly decreased by 29 and 16%, respectively, compared to control rats.

## Discussion

It is now universally accepted that mitochondria are a dynamic network of organelles, continuously modulating their functions and morphology to meet the needs of the cell. This phenomenon is particularly relevant in conditions of oxidative stress. Metabolically active compounds may alter mitochondrial activities and may consequently induce a condition of oxidative stress. Among these compounds are the hormones and, in particular, the THs that stimulate, among others, β-oxidation of fatty acids (25) and cellular respiration and that increase ROS production.

ROS may alter mitochondrial components such as enzymes, carriers, respiratory chain components, and in particular mtDNA. However, cells possess homeostatic mechanisms able to counteract these aggressive molecular species and consequently to maintain the normal cellular functions. Generally, the quality control mechanisms of the mitochondria and its genome can be organized into organellar (fusion-fission, autophagy/mitophagy) and DNA (DNA replication/transcription, DNA repair) maintenance programmes (26). The involvement of autophagy/mitophagy in the action of T3 has been recently investigated in studies by the group of Sinha et al. (27) and Sinha and Yen (28). Studies of the effects of T3 on mtDNA damage and related repair mechanisms are scarce, and there are no studies comparing the effects of T3 and of a metabolically active metabolite such as T2 on mtDNA damage and repair. To our knowledge, this study for the first time evaluates and compares the effects of either T3 or T2 on mtDNA damage and repair.

In the present investigation, we considered two pathways involved in preserving mitochondrial integrity: (i) mechanisms of repair and (ii) mitochondrial biogenesis/turnover that could lead to forming new, less damaged, organelles. This rationale could point to a possible different mechanism activated by the two iodothyronines. Indeed, from our data, it is evident that while T2 principally affects the BER pathways, T3 principally involves a mechanism leading to an increase in mitochondrial biogenesis. The data showing an increase of POLG expression in the case of T2 and an increase of PGC-1α and mtDNA content in the case of T3 validate this idea. Thus, T2 principally acts through the mechanism that directly repairs the lesions, while T3 inducing an activation of mitochondrial biogenesis, leads the cell to have a mitochondrial population enriched in new, less damaged, mitochondria. Indeed, the oxidative stress induced by T3 administration causes DNA damage, as suggested by the systemic 8-OHdG levels, but in the liver mtDNA lesions were reduced because of the increase in the number of new, less damaged mitochondria. In contrast, T2 treatment leads to a direct repair of the lesions with consequently less damage of DNA and the lack of an increase in the 8-OHdG serum level is in line with this.

**Figure 6:**
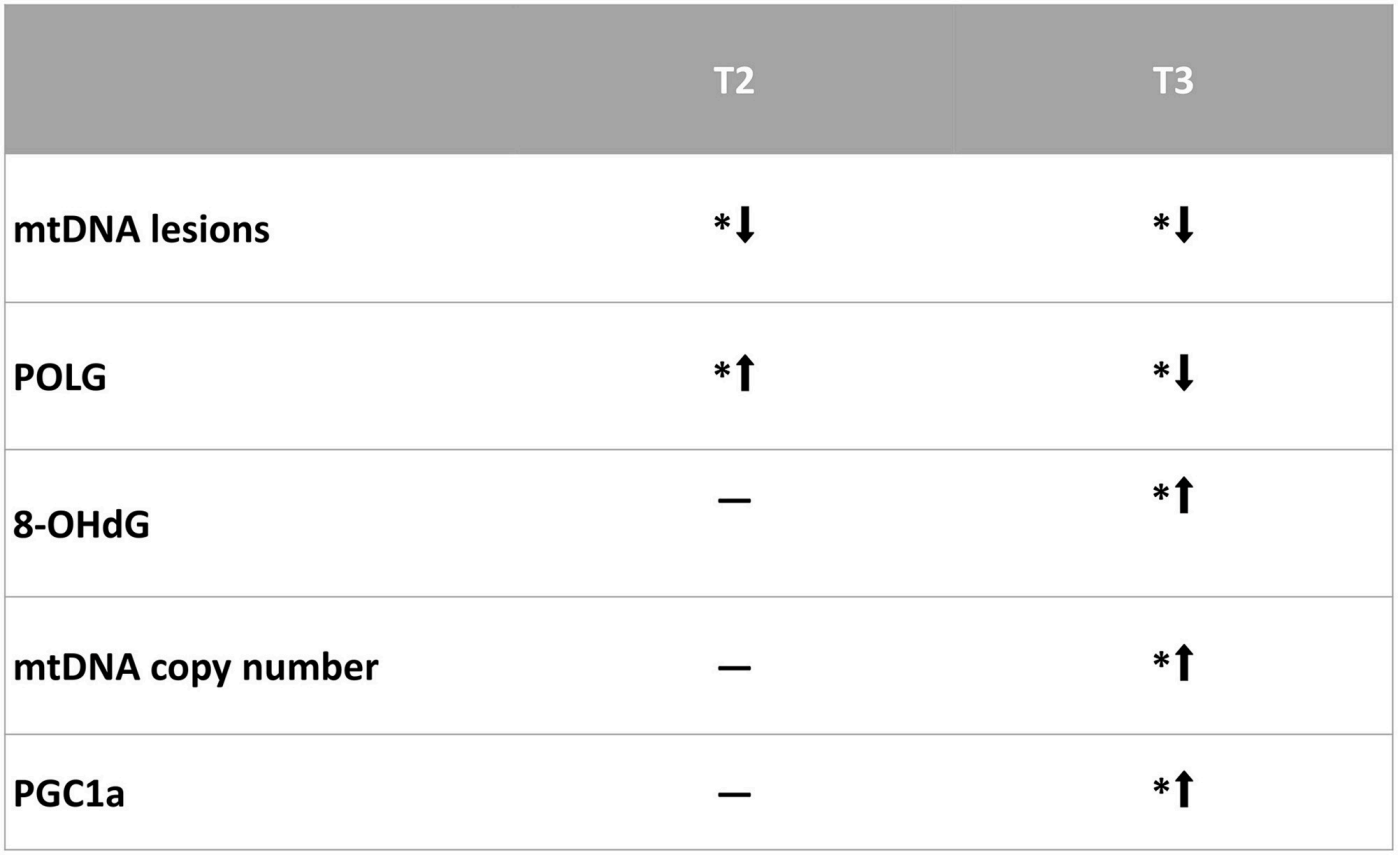
Representative scheme of the results obtained in this study. ^*^p < 0.05 vs. N rats. N: control; N+T2: rats receiving T2; N+T3: rats receiving T3.

In conclusion, both T3 and T2 are capable of inducing protective effects against mtDNA oxidative stress, but they affect different pathways. In the present study, we did not investigate the possible involvement of other homeostatic mechanisms possibly counteracting the oxidative stress, such as autophagy. However, this aspect has been recently investigated by Iannucci et al. (29), demonstrating that in the liver of HFD-treated rats, the administration of either T3 or T2 affected the autophagy mechanisms, but it was noteworthy that T_3_ induced mitophagy and mitochondrial biogenesis, whereas T_2_ did not. These data are in agreement with those reported in the present study. The main results of this study can be illustrated by the scheme in Figure 6.

## Author Contributions

FC, RS, FG, and AnL conceived and designed research. FC, RS, GP, and PL: performed experiments. FC and RS analyzed data, prepared figures, and drafted manuscript. FC, RS, PdL, ES, AsL, MM, FG, and AnL interpreted results of experiments. FC, RS, GP, PL, PdL, ES, AsL, MM, FG, and AnL edited and revised manuscript and approved final version of manuscript.

### Conflict of Interest Statement

The authors declare that the research was conducted in the absence of any commercial or financial relationships that could be construed as a potential conflict of interest.
